# Promote equal access to COVID19 vaccination: strategies of the Local Authority Toscana SudEst, Italy

**DOI:** 10.1093/eurpub/ckac131.122

**Published:** 2022-10-25

**Authors:** D Marconi, S Magi, C Cotoloni, C Moscatelli, S Arniani, F Nisticò, N Nante, G Messina

**Affiliations:** 1 Post Graduate School of Public Health, University of Siena, Siena, Italy; 2 Director of Valdarno Aretino Health District, Azienda USL Toscana Sud-Est, Arezzo, Italy; 3 Department of Territorial Health, Azienda USL Toscana Sud-Est, Grosseto, Italy; 4 Department of Health Promotion, Azienda USL Toscana Sud-Est, Siena, Italy; 5 Epidemiology Unit, Department of Prevention, Azienda USL Toscana Sud-Est, Arezzo, Italy; 6 Department of Molecular and Developmental Medicine, University of Siena, Siena, Italy

## Abstract

**Background:**

In the area of the Local Health Authority Toscana Sud-Est (LHA) 13,5% of residents are foreigners. We aim to assess the impact of our intervention to COVID-19 vaccination coverage.

**Methods:**

Since summer 2021, LHA has promoted vaccination sessions dedicated to foreign residents with free walk-in access, multilingual forms, flyers and TV interventions, cultural mediators and trained healthcare workers. We collected data about vaccination status of residents (28 December 2020-31 January 2022) and we analysed them using the software STATA to assess vaccine coverage by nationality and the effectiveness of our intervention. The results were adjusted for age and sex. We set significance level at p < 0.05.

**Results:**

On 31 July 2021, 78% of Italian residents (N = 685289) had received the first dose of vaccine, compared to only 43% of foreign residents (N = 106370). There was a 35% gap. On 31 January 2022, after our intervention, 89% of Italian residents and 71% of foreign residents had received the first dose of the vaccine. The gap was 18%. On 31 January 2022, 50% of residents of all nationalities had received two doses of the vaccine. A significant difference between Italian and foreign residents is still observed after adjustment for age and sex (OR 0.41 95% IC 0.40-0.41). Vaccination adherence is lower in females than males, for both Italian (OR 0.90 0.89-0.91) and foreign residents (OR 0.82 0.79-0.84). This is accentuated within some ethnic groups: Macedonians, Kosovars, Pakistanis.

**Conclusions:**

The creation of dedicated service guaranteed to reach a high vaccination coverage in all the nationalities and to reduce the gap between host and foreign residents. In foreigners it is lower than in the hosts, so it is necessary to investigate possible cultural factors that may influence hesitancy. A lower vaccination coverage in females, especially in foreigners, may be due to an inferior participation in social and working life as a consequence of the gender gap.

**Key messages:**

• The creation of dedicated interventions guaranteed to achieve high vaccination coverage in all nationalities.

• A lower vaccination adherence in females than males, especially in foreigners, may be due to an inferior participation in social and working life as a consequence of the gender gap.

